# Ladybird Beetle Diversity in Natural and Human-Modified Habitats in the San Cristóbal Island, Galapagos, Ecuador

**DOI:** 10.3390/insects15090725

**Published:** 2024-09-20

**Authors:** Emilia Peñaherrera-Romero, Ariel Guerrero-Campoverde, María P. Rueda-Rodríguez, Mateo Dávila-Játiva, Daniel Die-Morejón, Mariela Domínguez-Trujillo, Tomás Guerrero-Molina, Emilio Vélez-Darquea, Diego F. Cisneros-Heredia

**Affiliations:** 1 Laboratorio de Zoología Terrestre, Instituto de Biodiversidad Tropical IBIOTROP, Colegio de Ciencias Biológicas y Ambientales, Universidad San Francisco de Quito USFQ, Quito 170901, Ecuador; epenaherrera@usfq.edu.ec (E.P.-R.);; 2 Extensión GAIAS Galápagos, Universidad San Francisco de Quito USFQ, Puerto Baquerizo Moreno, San Cristóbal 200101, Ecuador; 3 Galápagos Science Center, Universidad San Francisco de Quito USFQ and University of North Carolina at Chapel Hill UNC, Puerto Baquerizo Moreno, San Cristóbal 200101, Ecuador; 4 Instituto Nacional de Biodiversidad INABIO, Quito 170506, Ecuador

**Keywords:** Galapagos, insect, non-native species, new records, Coccinellidae

## Abstract

**Simple Summary:**

This study explores the diversity of ladybird beetles on San Cristóbal Island, Galápagos Archipelago. We found nineteen species, including four already known and nine new ones reported for the first time on the island. Our research showed that endemic species, unique to the Galápagos, are rare and primarily found in native forests, highlighting their need for protection. The native species *Cycloneda sanguinea* was the most common and adaptable. Non-native species, like *Cheilomenes sexmaculata*, were found across all disturbed areas, including urban and agricultural zonas, and may be spreading into natural habitats. This information is crucial for conserving the Galápagos’ delicate ecosystems by emphasising the importance of monitoring and managing native and non-native insect species to preserve local biodiversity and prevent adverse environmental impacts.

**Abstract:**

This study investigates the species richness and distribution of ladybird beetles (Coccinellidae) across various habitats on San Cristóbal Island in the Galápagos Archipelago, Ecuador. Through extensive field surveys, we catalogued nineteen species, including four previously known species (two endemics, *Psyllobora bisigma* and *Scymnobius scalesius*, and two natives, *Cycloneda sanguinea* and *Tenuisvalvae bromelicola*). We also identified nine possibly native species reported for the first time in the Galapagos islands in this study or correspond to the first voucher specimens for the island. We collected three previously reported non-native species: *Cheilomenes sexmaculata*, *Novius cardinalis*, and *Paraneda guticollis*. Three species belonging to the genera *Stethorus*, *Calloeneis*, and *Delphastus* remain undetermined, pending further taxonomic analyses. Our findings reveal a rich and complex community with notable differences in species abundance and habitat preference. Endemic species were found to be particularly scarce and restricted mainly to crops undergoing forest regeneration and deciduous forests, emphasising their vulnerability and specialised habitat requirements. The native *Cycloneda sanguinea* emerged as the most prevalent species, exhibiting broad ecological adaptability. Non-native species, like *Cheilomenes sexmaculata*, were predominantly found in disturbed habitats, with some showing early signs of spreading into more natural environments, raising concerns about their potential impact on local biodiversity. These findings contribute valuable knowledge to understanding Coccinellidae diversity on San Cristóbal Island and highlight the importance of continued monitoring, particularly in the face of ongoing environmental change and the introduction of non-native species. This study underscores the need for targeted conservation efforts to protect the unique and fragile ecosystems of the Galápagos Archipelago.

## 1. Introduction

Insects, Earth’s most abundant group of animals, play fundamental roles in ecosystems. Their megadiversity translates into a remarkable range of ecological functions, including roles as pollinators, decomposers, soil engineers, and key members of most trophic matrices, with some species acting as pest controllers in human-made ecosystems [1,2,3,4]. Due to their isolation and unique evolutionary trajectories, insects in island ecosystems may take on even more critical roles than in mainland environments [1,2]. Island floras often have limited pollinators and may rely heavily on endemic insects, leading to the co-evolution of specialised pollination strategies [2,5]. In addition, the absence of top predators found on the mainland can elevate insects to keystone species across island trophic matrices, exerting a disproportionate influence on the overall health and functioning of island ecosystems [5,6].

In recent decades, insect decline has been reported as a worldwide phenomenon with severe cascading effects, disrupting food webs, hindering plant reproduction, impacting human health, and ultimately compromising the health of entire environments [7,8,9,10]. Global declines of insect populations [11,12,13] cast their shadow over island ecosystems, and these isolated communities may be even more vulnerable to catastrophic insect declines. Island insects, often with limited geographical ranges and specialised ecological roles, face a higher risk of extinction if their habitat or food sources disappear [12,13]. The factors driving global insect declines—habitat loss, environmental pollution, introduced species, and climate changes—are likely to have amplified effects on islands, which typically have fewer refugees and more fragile ecological dynamics [14,15,16,17,18,19]. Understanding insect communities in islands is crucial for identifying threats and implementing targeted conservation strategies. In addition to global declines, most oceanic islands have experienced significant shifts in insect assemblages due to the introductions of non-native species and the displacement of native insects [15,20].

Coccinellidae is a charismatic and ecologically important family of beetles, commonly known as ladybird beetles or ladybugs, found worldwide, with over 6000 species described to date [21,22]. Although some ladybird beetle species are easily recognised by their colourful elytra, the family has a remarkable diversity of body shapes, sizes, and colour patterns [15,23]. Many species of ladybird beetles are recognised for their role as voracious predators of agricultural pests. However, the trophic strategies of Coccinellidae are diverse, with several species consuming fungi or plant material. Due to the role of many predatory species as biological control agents, several species of ladybird beetles have been deliberately translocated worldwide. A classic biological control success example is the intentional introduction of the Vedalia ladybird beetle (*Novius cardinalis*) to the Galapagos Archipelago, which effectively controlled the invasive cottony cushion scale (*Icerya purchasi*) [24,25,26].

Few studies have documented the diversity of ladybird beetles in the Galapagos Archipelago [27,28,29,30,31,32]. Peck [31] reported twelve species based on a long-term research project focused on documenting and analysing insect faunas of the archipelago. Peck [31] considered ten species as native to the Galapagos, with six being endemic (marked with an asterisk): Cycloneda galapagoensis*, *C. sanguinea*, *Diomus anthony*, Olla hageni*, O. lacrimosa*, Psyllobora bisigma*, Scymnobius scalesius*, *S. galapagoensis*, *Tenuisvalvae bromelicola*, and an undescribed species of Pentilia*. Additionally, two non-native species were identified: *Coccidophilus* sp. and *Novius cardinalis*. Subsequent studies have had narrower geographical and methodological scopes or were based on accidental or unvouchered records. Cañarte Bermudez [33] documented *Cycloneda sanguinea* and three non-native species from agricultural areas in San Cristóbal and Santa Cruz Islands: *Cheilomenes sexmaculata* (as *Cheilomenes* sp.), *Hippodamia convergens*, and *Stethorus* sp. Carvajal Román et al. [34] reported *Paraneda guticollis* without specific localities. *Hyperaspis onerata* was determined on the “Coccinellidae de Ecuador” website [35] based on participative science observations from San Cristóbal Island uploaded to iNaturalist. *Diomus tucumanus*, *Eriopis connexa*, *Psyllobora confluens*, and *Serratitibia loreto* were reported as intercepted on aeroplanes based on an unpublished report of the Agency of Regulation and Control of the Biosecurity and Quarantine for Galapagos ABG [36,37,38,39].

Herein, we present information on the ladybird beetle community of the San Cristóbal Island, Galapagos Archipelago, based on surveys conducted in 2019, 2022, and 2023 across different natural and human-modified landscapes.

## 2. Materials and Methods

### 2.1. Study Area

The Galapagos islands, an archipelago of volcanic origin, are located 930 km west of mainland Ecuador in the eastern Pacific Ocean. The archipelago consists of 19 main islands (>1 km^2^) and more than 100 islets and rocks [40,41]. Most of the insular land area (96.77%) falls under the protection of the Galapagos National Park. The remaining areas, not part of the national park, are those destined for human use, where human settlements, agriculture, and other activities are concentrated. Out of the 19 islands of the archipelago, only Santa Cruz, San Cristóbal, Isabela, and Floreana are inhabited by humans [42]. San Cristóbal Island, the archipelago’s easternmost and geologically oldest island, is the fifth largest, with an area of 558 km^2^ [41]. The lowlands of San Cristóbal are dry and warm, with a narrow belt of littoral vegetation and a wide area covered by deciduous forests and shrubland. With increasing altitude, the habitats become more mesic, and a transition zone appears, covered by seasonal evergreen shrubland and forests with taller trees, denser canopies, and wetter conditions. This transition zone has a plant community mixing species from the lower and higher zones. In the highlands, moist conditions allow for zones of increased humidity and denser vegetation dominated by evergreen forests and shrubland. Above the regional tree line, a treeless humid tallgrass zone is dominated by sedges and ferns [42,43,44,45]. Human settlement on San Cristóbal Island began in the second half of the 19th century, leading to significant anthropogenic land cover changes. Currently, 17% of the island’s land area comprises human-modified landscapes, including two urban settlements (Puerto Baquerizo Moreno in the dry lowlands and El Progreso in the humid highlands) and the agricultural regions that have largely occupied areas formerly covered by seasonal evergreen and evergreen shrubland and forests [45,45,46]. Ecosystem and vegetation typologies herein follow those proposed by Rivas-Torres et al. [42] and Laso et al. [45].

### 2.2. Data Collection and Analysis

**Figure 1:**
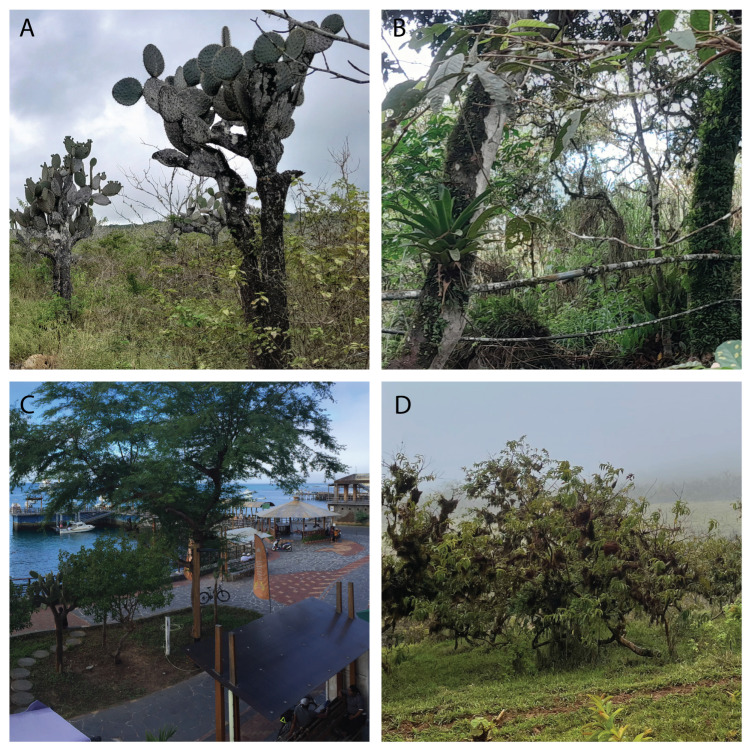
Some of the ecosystems surveyed in San Cristóbal Island. (**A**) Deciduous forest, (**B**) seasonal evergreen forest, (**C**) urban, (**D**) agricultural.

**Figure 2:**
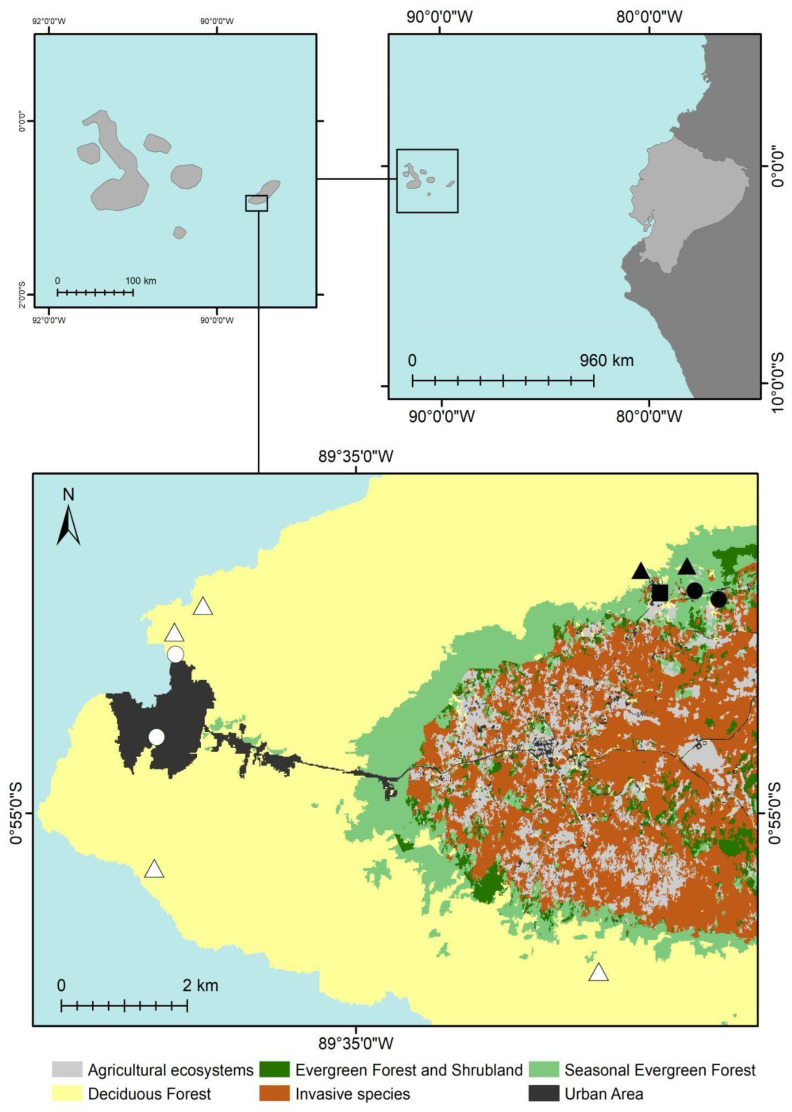
Map showing the localities explored during our surveys for ladybird beetles in San Cristóbal Island, Galapagos Archipelago, Ecuador. White circles = urban green areas, white triangles = deciduous forest, black circles = silvopasture, black triangles = seasonal evergreen forests mixed with blackberry and supirosa, black square = permanent crops undergoing native forest regeneration.

We conducted standardised surveys in twelve localities in the lowlands and highlands of San Cristóbal Islands, covering two human-modified landscapes (urban and agricultural) and two natural ecosystems (deciduous and seasonal evergreen forests) (Table 1, Figure 1 and Figure 2). Surveys were carried out by one to three researchers in the morning (08 h 30–11 h 00), afternoon (14 h 30–17 h 00), and evening (20 h 00–23 h 00), excluding days of heavy rain. We meticulously looked for beetles at each locality through visual surveys along a 200 m transect. Surveys were conducted in 2019 (30 June–3 July, sampling effort 33 person h; 21 July–9 August, 168 person h), 2021 (9–16 May, 41 person h; 4–6 October, 14 person h), 2022 (14–16 February, 15 person h; 15–21 August, 56 person h), and 2023 (12–29 July, 144 person h), totalling a sampling effort of 471 person h. In addition, we obtained occurrence data for individuals of Coccinellidae from San Cristóbal Island updated to iNaturalist https://www.inaturalist.org (accessed on 21 March 2024), a citizen science platform by the California Academy of Science and National Geographic. Data search and extraction were conducted in March 2024. For each occurrence point, we compiled geographic data and all other associated information, and localities were reviewed and validated individually, following protocols described by Cisneros-Heredia and Peñaherrera-Romero [47] and Cisneros-Heredia et al. [48]. All geographic records of Coccinellidae from San Cristóbal Island reported in this paper are available at https://doi.org/10.5281/zenodo.11173701 (accessed on 21 March 2024).

We did not collect every individual of common species easily identifiable based on their morphology and colouration (i.e., *Cycloneda sanguinea*, *Cheilomenes sexmaculata*, *Pentilia guticollis*). Still, all uncollected individuals were registered in our survey database. Individuals of all other taxa were preserved as voucher specimens. Specimens were collected by hand, euthanised with 75% ethanol, and transferred to the Laboratorio de Zoología Terrestre, Universidad San Francisco de Quito, Quito, Ecuador, where they were dry pinned. Specimens were examined under a Nikon SMZ2745 stereomicroscope (Nikon Corporation, Tokyo, Japan). Specimens were identified to the lowest taxonomic level using the taxonomic keys by Gordon and Chapin [49], Gordon and González [32,50], González and Almeida [51], Gordon [52], Gordon, González, and Hanley [53,54], and González, Hanley, and Gordon [55]. We obtained compound images by stacking a series of photographs taken at different depths using an Olympus DP73 digital camera (Olympus Corporation, Tokyo, Japan) attached to an Olympus SZX16 stereomicroscope (Olympus Corporation, Tokyo, Japan) and processing the photographs with Helicon Focus 8 (Helicon Soft Ltd., Kharkiv, Ukraine). For quantitative analysis and to understand the contribution of a species to the community of each habitat, we calculated its relative abundance by dividing the total number of individuals of each species in each habitat by the total number of specimens found in that habitat.

We classified the species’ origin according to their arrival in the Galapagos islands. Native species originated in the archipelago or arrived by their own means from another area where they were native due to their adaptations for dispersal and survival across routes that are not strictly natural barriers. Endemic species are a specific type of native taxa that evolved from a founder species that arrived in the archipelago and are usually differentiated due to long-term isolation, resulting in genetic divergence. Endemic species are unique to the Galapagos and, in some cases, to specific islands within the archipelago. Non-native species have arrived from an area where they are non-native or came from their native range by extrinsic dispersal mechanisms, which provide specific conditions that allow these taxa to disperse in the same timeframe across environments that would otherwise be solid natural barriers [56]. All species reported for the first time after Peck [31] are considered possibly native unless they show a strong association with human-modified environments or have been detected in areas thoroughly surveyed in the past, in which case they are regarded as possibly non-native [57]. Due to the limited knowledge and taxonomic work on the ladybird beetles of western South America, there is uncertainty as to whether species first reported as distributed in areas west of the Andes and more recently in the Galapagos could be native to the islands and have been overlooked by previous expeditions on San Cristóbal, considering that much of the research on ladybirds and insects in general in the archipelago has focused on other islands, such as Santa Cruz and Isabela [15,31,58]. The origin of taxa not identified at the species level is reported as undetermined.

## 3. Results

**Figure 3:**
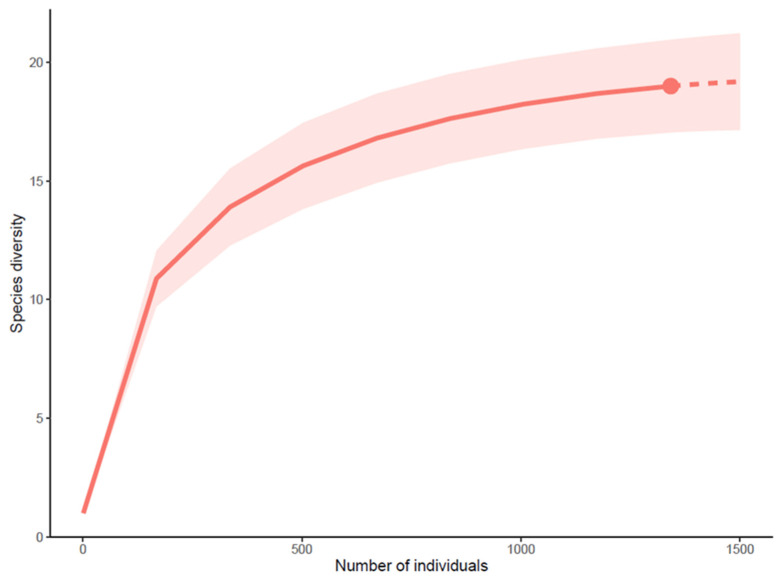
Species accumulation rarefaction curve, with the red dot indicating the extent covered by our surveys.

During our study in San Cristóbal Island, we documented 1359 individuals representing 19 species of ladybird beetles, including two endemic species, *Psyllobora bisigma* and *Scymnobius scalesius*, and two native species, *Cycloneda sanguinea* and *Tenuisvalvae bromelicola*, previously recorded in the island. We also recorded nine possibly native species reported for the first time in the Galapagos islands in this study or that correspond to the first voucher specimens for the island (Table 2). We collected three previously reported non-native species: *Cheilomenes sexmaculata*, *Novius cardinalis*, and *Paraneda guticollis*. Three species belonging to the genera *Stethorus*, *Calloeneis*, and *Delphastus* remain undetermined pending further taxonomic studies (Table 2). Despite survey efforts, the species accumulation curve, while almost flat, indicates that we have not yet reached a plateau, suggesting that more species remain unrecorded (Figure 3). We did not collect species of the four species previously reported in San Cristóbal Island: *Diomus* sp., *Coccidophilus* sp., *Scymnobius galapagoensis*, and *Hippodamia convergens*; however, there is a single record of a *Diomus* from San Cristóbal in iNaturalist [59].

**Figure 4:**
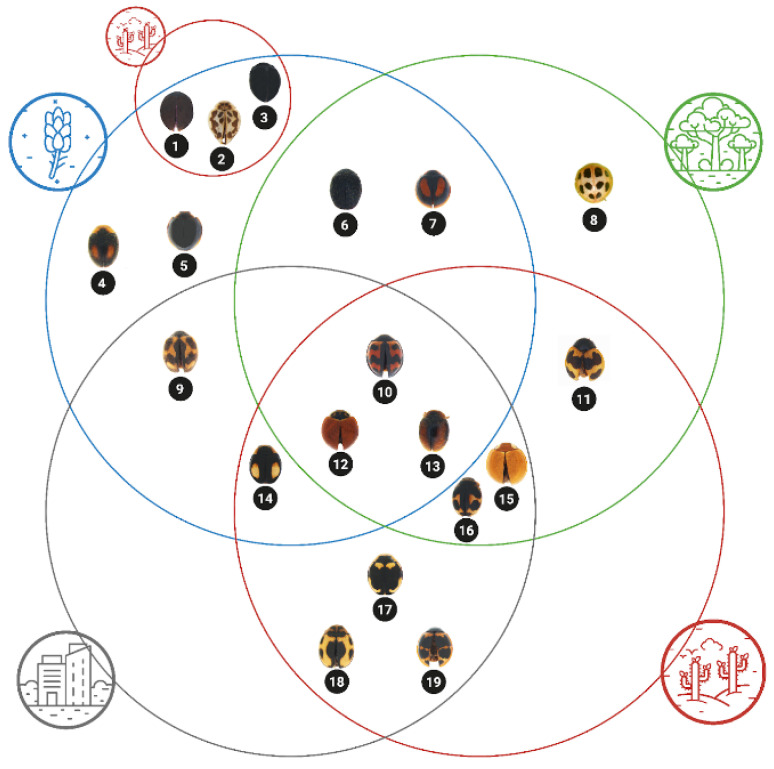
Species presence across the four surveyed ecosystems in San Cristóbal Island, Galapagos. Coloured circles represent each ecosystem (urban = grey, agricultural = blue, seasonal evergreen forest = green, and deciduous forest = red). Species found in the intersecting areas between the circles correspond to those shared between the ecosystems represented by the circles. Three species found in deciduous forests and agricultural areas are shown within a small red circle intersecting the blue circle. Species represented are as follows: (1) *Calloeneis* sp., (2) *Psyllobora bisigma*, (3) *Delphastus* sp., (4) *Scymnobius scalesius*, (5) *Pentilia bernadette*, (6) *Stethorus* sp., (7) *Pentilia chelsea*, (8) *Zagreus constantini*, (9) *Novius cardinalis*, (10) *Cheilomenes sexmaculata*, (11) *Zagreus cornejoi*, (12) *Cycloneda sanguinea*, (13) *Scymnobius ecuadoricus*, (14) *Hyperaspis esmeraldas*, (15) *Paraneda guticollis*, (16) *Hyperaspis festiva*, (17) *Tenuisvalvae bromelicola*, (18) *Hyperaspis onerata*, (19) *Zagreus decempunctatus*.

Both endemic species were rare and recorded in a few ecosystems, primarily permanent crops undergoing forest regeneration and deciduous forests. The native *Cycloneda sanguinea* was the most abundant across all ecosystems, while the native *Tenuisvalvae bromelicola* was rare and found in only two ecosystems: deciduous forests and urban green areas. Among the possibly native species, most species were rare and heterogeneously found across different ecosystems (Figure 4). However, *S. ecuadoricus* was found in all ecosystems, with a higher abundance in deciduous forests. *Zagreus cornejoi* was present in urban areas and deciduous forests, but it was only abundant in the latter. *Zagreus constantini* and *Pentilia bernadette* were singleton species from evergreen forests and agricultural areas, respectively. The non-native species, *Cheilomenes sexmaculata*, was present in all ecosystems and was more abundant in agricultural and urban areas. *Paraneda guticollis* was found in deciduous forests and agricultural and urban areas. Still, it was only abundant in urban areas, thus suggesting that it is non-native and could be expanding towards natural habitats.

*Species Accounts*

**Family: Coccinellidae**
**Subfamily: Coccinellinae**
**Tribe: Stethorini**

**Figure 5:**
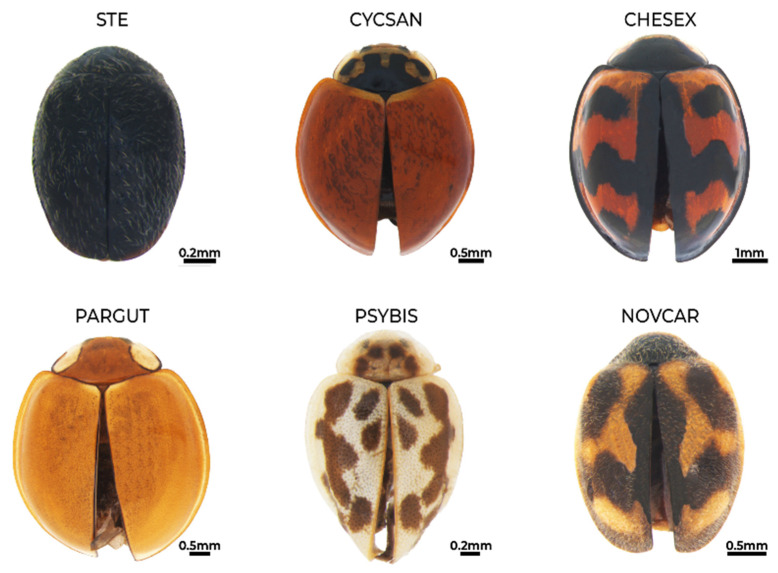
Habitus of *Stethorus* sp. (STE), *Cycloneda sanguinea* (CYCSAN), *Cheilomenes sexmaculata* (CHESEX), *Paraneda guticollis* (PARGUT), *Psyllobora bisigma* (PSYBIS), and *Novius cardinalis* (NOVCAR).

*Stethorus* sp. (Figure 5)
**Status in Galapagos:** Undetermined.
**Global distribution:** Genus is widespread in America, Asia, Africa, Australia, and Europe [49].
**Distribution in the Galapagos islands:** Santa Cruz [33] and San Cristóbal. This is the first report of this genus for San Cristóbal.
**Ecosystems in San Cristóbal Island:** Permanent crops (coffee and guava) undergoing native forest regeneration and seasonal evergreen forest mixed with blackberry and supirosa.
**Diagnosis:** Small <2 mm. Species of this genus are characterised by having a black or dark brown ovoid convex body with dense pubescence of variable size and fine or thick perforated texture. Antennae, legs, and mouthparts are yellow brown, and post-coxal lines are closed [49]. Species-level identification requires examining male genitalia.
**Remarks:** Cañarte Bermúdez et al. [33] reported the genus in Santa Cruz Island but without a description or diagnostic details. It may correspond to this species. Further research, including genitalia examination, is required to identify the species present in the Galapagos. There are no observations of this genus from the Galápagos in iNaturalist.
**Voucher specimens:** ECUADOR • one specimen; Galapagos province, San Cristóbal Island, Hacienda Tranquila, regeneration site; −0.886647, −89.539828; 392 m alt.; 30 July 2019; E. Peñaherrera-Romero and E. Cadena leg.; ZSFQ • one specimen; Galapagos province, San Cristóbal Island, Galapagos National Park patch 1; −0.883996, −89.539673; 365 m alt.; 29 July 2019; E. Peñaherrera-Romero and E. Cadena leg.; ZSFQ.

**Tribe: Coccinellini**

*Cheilomenes sexmaculata* (Fabricius, 1781) [60] (Figure 5)
**Status in Galapagos:** Non-native.
**Global distribution:** Native to East, South, and Southeast Asia, Australia, New Guinea, and the western Pacific Islands [61,62]. In South America, extra-range records of *C. sexmaculata* have been reported from Venezuela, Colombia, Ecuador, Peru, and Chile [63,64,65,66,67,68].
**Distribution in the Galapagos islands:** Floreana, Isabela, San Cristóbal, Santa Cruz, and Santiago [33,69,70].
**Ecosystems in San Cristóbal Island:** Urban green areas, silvopasture, permanent crops (coffee and guava) undergoing forest regeneration, seasonal evergreen forest mixed with blackberry and supirosa, and deciduous forests.
**Diagnosis:** Size 4–5 mm. Ivory to dark brown head, a dark brown pronotum that sometimes has white spots on the anterior border, ground colour of the elytra is usually orange with variable black spots, and elytra are dark brown to black with orange to red spots on anterior margins and sometimes on the posterior part of the elytra [68].
**Remarks:** It was the third most abundant ladybird beetle species in our surveys, after *Cycloneda sanguinea* and *Paraneda guticollis*. Cañarte Bermudez [33] first recorded this species in crops on San Cristóbal and Santa Cruz islands in 2016–2017. An early expedition by our team found it in urban areas in 2018 (F. Carrera and D. F. Cisneros-Heredia, see iNaturalist). The species has recently expanded across the islands and has the potential to become an invasive species.
**Voucher specimens:** ECUADOR • nineteen specimens; Galapagos province, San Cristóbal Island, Hacienda Tranquila, agricultural lands; −0.887872, −89.539682; 384 m alt.; 2 and 4 August 2019; E. Peñaherrera-Romero and E. Cadena leg.; ZSFQ • two specimens; Galapagos province, San Cristóbal Island, Hacienda Tranquila, agricultural lands; −0.887872, −89.539682; 384 m alt.; 18 February 2022; D.F. Cisneros-Heredia leg.; ZSFQ • ten specimens; Galapagos province, San Cristóbal Island, Hacienda Tranquila, regeneration site; −0.886647, −89.539828; 392 m alt.; 27 and 30 July 2019; E. Peñaherrera-Romero and E. Cadena leg.; ZSFQ • five specimens; Galapagos province, San Cristóbal Island, Hacienda Tranquila, regeneration site; −0.886647, −89.539828; 392 m alt.; 17 August 2022; E. Peñaherrera-Romero, D. Die-Morejón, A. Guerrero-Campoverde and T. Guerrero-Molina leg.; ZSFQ • twelve specimens; Galapagos province, San Cristóbal Island, Hacienda Tranquila, regeneration site; −0.886647, −89.539828; 392 m alt.; 15 July 2023; E. Peñaherrera-Romero, M.P. Rueda-Rodríguez, and E. Vélez-Darquea leg.; ZSFQ •nine specimens; Galapagos province, San Cristóbal Island, Playa Mann and Environmental Interpretation Center; −0.894897, −89.60904; 9 m alt.; 16 and 17 July 2019; E. Peñaherrera-Romero and E. Cadena leg.; ZSFQ • one specimen; Galapagos province, San Cristóbal Island, Playa Mann and Environmental Interpretation Center; −0.894897, −89.60904; 9 m alt.; 15 August 2022; E. Peñaherrera-Romero, D. Die-Morejón, A. Guerrero-Campoverde and T. Guerrero-Molina leg.; ZSFQ • nine specimens; Galapagos province, San Cristóbal Island, Playa Mann and Environmental Interpretation Center; −0.894897, −89.60904; 9 m alt.; 13 and 25 July 2023; E. Peñaherrera-Romero, M.P. Rueda-Rodríguez, and E. Vélez-Darquea leg.; ZSFQ • twelve specimens; Galapagos province, San Cristóbal Island, Encañada ravine; −0.905924, −890.612054; 13 m alt.; 22 and 25 July 2019; E. Peñaherrera-Romero and E. Cadena leg.; ZSFQ • one specimen; Galapagos province, San Cristóbal Island, Galapagos National Park patch 1; −0.883996, −89.539673; 365 m alt.; 28 July 2019; E. Peñaherrera-Romero and E. Cadena leg.; ZSFQ • four specimens; Galapagos province, San Cristóbal Island, Galapagos National Park patch 2; −0.88374, −89.539804; 365 m alt.; 31 July and 1 August 2019; E. Peñaherrera-Romero and E. Cadena leg.; ZSFQ • thirty-seven specimens; Galapagos province, San Cristóbal Island, Puerto Baquerizo Moreno town; −0.904573, −89.611143; 13 m alt.; 21 July 201; E. Peñaherrera-Romero and E. Cadena leg.; ZSFQ • nine specimens; Galapagos province, San Cristóbal Island, Baquerizo Beach trail; −0.888899, −79.607597; 40 m alt.; 19 July 2019; E. Peñaherrera-Romero and E. Cadena, leg.; ZSFQ.

**Figure 6:**
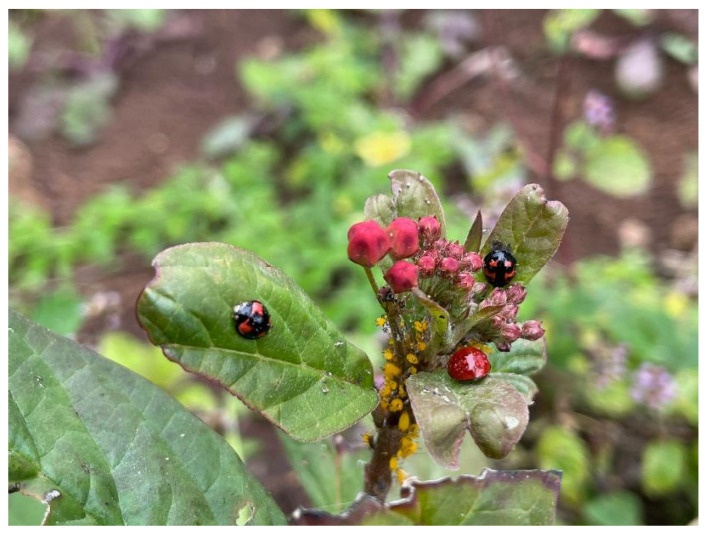
*Cycloneda sanguinea* and *Cheilomenes sexmaculata* predating on *Aphis nerii*.

*Cycloneda sanguinea* (Linneus, 1763) [71] (Figure 5)
**Status in Galapagos:** Native.
**Global distribution:** Widespread from southern USA to Argentina [72].
**Distribution in the Galapagos islands:** Española, Fernandina, Floreana, Genovesa, Isabela, Marchena, Pinta, Pinzón, Rábida, Santa Cruz, San Cristóbal, Santiago, and Wolf [31].
**Ecosystems in San Cristóbal Island:** Urban green areas, agricultural areas, deciduous forests, and seasonal evergreen forests mixed with blackberry and supirosa.
**Diagnosis:** Size 3–6 mm. Easily diagnosed by its semicircular shape with flat elytra coloured orange, red, or brick red. Legs, mouthparts, and abdomen are black. The head and pronotum are black with two round white spots [72].
**Remarks:** The most abundant species of ladybird beetle in San Cristóbal Island in our surveys and also based on iNaturalist observations. The species was regularly associated with *Cheilomenes sexmaculata* and *Paraneda guticollis* in urban and agricultural areas. *Cycloneda sanguinea* and *Cheilomenes sexmaculata* were commonly found together in highland agricultural lands, predating on milkweed aphids *Aphis nerii* (Figure 6).
**Voucher specimens:** ECUADOR • 123 specimens; Galapagos province, San Cristóbal Island, Playa Mann and Environmental Interpretation Center; −0.894897, −89.60904; 9 m alt; 16 July 2019; E. Peñaherrera-Romero and E. Cadena leg.; ZSFQ • 1 specimen; Galapagos province, San Cristóbal Island, Playa Mann and Environmental Interpretation Center; −0.894897, −89.60904; 9 m alt.; 15 August 2022; E. Peñaherrera-Romero, D. Die-Morejón, A. Guerrero-Campoverde and T. Guerrero-Molina leg.; ZSFQ • 46 specimens; Galapagos province, San Cristóbal Island, Playa Mann and Environmental Interpretation Center; −0.894897, −89.60904; 9 m alt.; 12, 13 and 25 July 2023; E. Peñaherrera-Romero, M.P. Rueda-Rodríguez, and E. Vélez-Darquea leg.; ZSFQ • 21 specimens; Galapagos province, San Cristóbal Island, Baquerizo Beach trail; −0.888899, −79.607597; 40 m alt.; 18 and 19 July 2019; E. Peñaherrera-Romero and E. Cadena leg.; ZSFQ • 12 specimens; Galapagos province, San Cristóbal Island, Baquerizo Beach trail; −0.888899, −79.607597; 40 m alt.; 23 and 25 July 2023; E. Peñaherrera-Romero, M.P. Rueda-Rodríguez, and E. Vélez-Darquea leg.; ZSFQ • 16 specimens; Galapagos province, San Cristóbal Island, Puerto Baquerizo Moreno town; −0.904573, −89.611143; 13 m alt.; 21 July 2019; E. Peñaherrera-Romero and E. Cadena leg.; ZSFQ • 13 specimens; Galapagos province, San Cristóbal Island, Encañada ravine; −0.905924, −89.612054; 13 m alt.; 22 and 25 July 2019; E. Peñaherrera-Romero and E. Cadena leg.; ZSFQ • 55 specimens; Galapagos province, San Cristóbal Island, Hacienda Tranquila, agricultural lands; −0.887872, −89.539682; 384 m alt.; 2 and 4 August 2019; E. Peñaherrera-Romero and E. Cadena leg.; ZSFQ • 8 specimens; Galapagos province, San Cristóbal Island, Hacienda Tranquila, agricultural lands; −0. 887872, −89.539682; 384 m alt.; 18 February 2022; D.F. Cisneros-Heredia leg.; ZSFQ • 160 specimens; Galapagos province, San Cristóbal Island, Hacienda Tranquila, regeneration site; −0.886647, −89.539828; 392 m alt.; 27 and 30 July 2019; E. Peñaherrera-Romero and E. Cadena leg.; ZSFQ • 47 specimens; Galapagos province, San Cristóbal Island, Hacienda Tranquila, regeneration site; −0.886647, −89.539828; 392 m alt.; 17 August 2022; E. Peñaherrera-Romero, D. Die-Morejón, A. Guerrero-Campoverde and T. Guerrero-Molina leg.; ZSFQ • 39 specimens; Galapagos province, San Cristóbal Island, Hacienda Tranquila, regeneration site; −0.886647, −89.539828; 392 m alt.; 14 and 15 July 2023; E. Peñaherrera-Romero, M.P. Rueda-Rodríguez, and E. Vélez-Darquea leg.; ZSFQ • 11 specimens; Galapagos province, San Cristóbal Island, Opuntias Beach trail; −0.932561, −89.584034; 40 m alt.; 23 and 24 July 2019; E. Peñaherrera-Romero and E. Cadena leg.; ZSFQ • 4 specimens; Galapagos province, San Cristóbal Island, Opuntias Beach trail; −0.932561, −89.584034; 40 m alt.; 16 August 2022; E. Peñaherrera-Romero, D. Die-Morejón, A. Guerrero-Campoverde and T. Guerrero-Molina leg.; ZSFQ • 1 specimen; Galapagos province, San Cristóbal Island, Opuntias Beach trail; −0.932561, −89.584034; 40 m alt.; 26 July 2023; E. Peñaherrera-Romero, M.P. Rueda-Rodríguez, and E. Vélez-Darquea leg.; ZSFQ • 80 specimens; Galapagos province, San Cristóbal Island, Galapagos National Park patch 1; −0.883996, −89.539673; 365 m alt.; 28 and 29 July 2019; E. Peñaherrera-Romero and E. Cadena leg.; ZSFQ • 36 specimens; Galapagos province, San Cristóbal Island, Galapagos National Park patch 2; −0.88374, −89.539804; 365 m alt.; 31 July and 1 August 2019; E. Peñaherrera-Romero and E. Cadena leg.; ZSFQ • 10 specimens; Galapagos province, San Cristóbal Island, Hacienda Tranquila, Risco de los Petreles; −0.887209, −89.531328; 500 m alt.; 18 August 2022; E. Peñaherrera-Romero, D. Die-Morejón, A. Guerrero-Campoverde and T. Guerrero-Molina leg.; ZSFQ • 3 specimens; Galapagos province, San Cristóbal Island, Lobería Beach; −0.923923, −89.614669; 5 m alt.; 19 August 2022; E. Peñaherrera-Romero, D. Die-Morejón, A. Guerrero-Campoverde and T. Guerrero-Molina leg.; ZSFQ.

*Paraneda guticollis* (Mulsant, 1850) [73] (Figure 5)
**Status in Galapagos:** Possibly non-native.
**Global distribution:** México, Ecuador, and Bolivia [74,75].
**Distribution in the Galapagos islands:** Floreana, Isabela, San Cristóbal, and Santa Cruz. Our specimens are the first vouchers reported for the Galapagos.
**Ecosystems in San Cristóbal Island:** We collected this species in the urban and periurban green areas of Puerto Baquerizo Moreno, permanent crops undergoing native forest regeneration and deciduous forests.
**Diagnosis:** Size 4–6 mm. Individuals with a round, convex body. Dark yellow head. Ochre yellow pronotum with two round beige spots at one end each, and these spots usually have a black border. Ochre yellow elytra with a black border that may not be present separating the elytra in the centre. The underside of the elytra is beige. Ochre yellow legs, antennae, and mouthparts. Without pubescence [73,75].
**Remarks:** This species is the most abundant ladybird beetle in urban areas. The species is regarded as possibly non-native because it has been recorded only in recent years on the archipelago [34] and it is most frequent in human-modified habitats, mainly urban areas. Its abundance and frequency have increased in recent years, and it has the potential to become an invasive species.
**Voucher specimens:** ECUADOR • 386 specimens; Galapagos province, San Cristóbal Island, Encañada ravine; −0.905924, −89.612054; 13 m alt.; 22 and 25 July 2019; E. Peñaherrera-Romero and E. Cadena leg.; ZSFQ • three specimens; Galapagos province, San Cristóbal Island, Puerto Baquerizo Moreno town; −0.904573, −89.611143; 13 m alt.; 21 July 2019; E. Peñaherrera-Romero and E. Cadena leg.; ZSFQ • four specimens; Galapagos province, San Cristóbal Island, Hacienda Tranquila, regeneration site, −0.886647, −89.539828; 392 m alt.; 17 February 2022; D.F. Cisneros-Heredia leg.; ZSFQ • one specimen; Galapagos province, San Cristóbal Island, Playa Mann and Environmental Interpretation Center; −0.894897, −89.60904; 9 m alt.; 15 August 2022; E. Peñaherrera-Romero, D. Die-Morejón, A. Guerrero-Campoverde and T. Guerrero-Molina leg.; ZSFQ • three specimens; Galapagos province, San Cristóbal Island, Playa Mann and Environmental Interpretation Center; −0.894897, −89.60904; 9 m alt.; 13 July 2023; E. Peñaherrera-Romero, M.P. Rueda-Rodríguez, and E. Vélez-Darquea leg.; ZSFQ • one specimen; Galapagos province, San Cristóbal Island, Baquerizo Beach trail; −0.888899, −79.607597; 40 m alt.; 19 July 2019; E. Peñaherrera-Romero and E. Cadena leg.; ZSFQ.

*Psyllobora bisigma* Van Dike, 1953 [73] (Figure 5)
**Status in Galapagos:** Endemic.
**Global distribution:** Found only in the Galapagos Archipelago [31,73].
**Distribution in the Galapagos islands:** Fernandina, Isabela, Pinta, San Cristóbal, Santa Cruz, and Santiago [31,73].
**Ecosystems in San Cristóbal Island:** Permanent crops (coffee and guava) undergoing native forest regeneration and deciduous forests.
**Diagnosis:** Size 2–3 mm. Elongated body at the lower margin. Light brown head. Beige pronotum with five light brown spots. Beige elytra with nine light brown-brown spots partially joined together. Light brown legs, antennae, and mouthparts. Punctured texture [73].
**Voucher specimens:** ECUADOR • one specimen; Galapagos province, San Cristóbal Island, Opuntias Beach trail; −0.932561, −89.584034; 40 m alt.; 24 July 2019; E. Peñaherrera-Romero and E. Cadena leg.; ZSFQ • nine specimens; Galapagos province, San Cristóbal Island, Hacienda Tranquila, regeneration site; −0.886647, −89.539828; 392 m alt.; 14 and 15 July 2023; E. Peñaherrera-Romero, M.P. Rueda-Rodríguez, and E. Vélez-Darquea leg.; ZSFQ.

**Tribe:** Noviini

*Novius cardinalis* (Mulsant, 1850) [73] (Figure 5)
**Status in Galapagos:** Non-native.
**Global distribution:** This is a cosmopolitan species of Australian origin introduced in some countries due to its success in biological pest control [25,76].
**Distribution in the Galapagos islands:** Baltra, Floreana, Isabela, Marchena, Pinta, Rábida, San Cristóbal, Santa Cruz, and Santiago [15].
**Ecosystems in San Cristóbal Island:** Urban green areas and permanent crops (coffee and guava) undergoing native forest regeneration.
**Diagnosis:** Size 2–4 mm. Circular body. Black head. Red pronotum with a semicircular black spot. Red elytra with four spots on each elytrum that join together, and a black spot extends along the central margin separating the elytra. The antennae, legs, and mouthparts are red. Golden pubescence [76].
**Remarks:** It was introduced in the Galapagos Archipelago in 2006 as a biocontrol agent of *Icerya purchasi*, cottony cushion scale [15]. Many observed specimens were not collected during our surveys.
**Voucher specimens:** ECUADOR • one specimen; Galapagos province, San Cristóbal Island, Hacienda Tranquila, agricultural lands; −0.886647, −89.539828; 392 m alt.; 30 July 2019; E. Peñaherrera-Romero and E. Cadena leg.; ZSFQ • one specimen; Galapagos province, San Cristóbal Island, Playa Mann and Environmental Interpretation Center; −0.894897, −89.60904; 9 m alt.; 25 July 2023; E. Peñaherrera-Romero, M.P. Rueda-Rodríguez, and E. Vélez-Darquea leg.; ZSFQ.

**Tribe: Scymnini**

**Figure 7:**
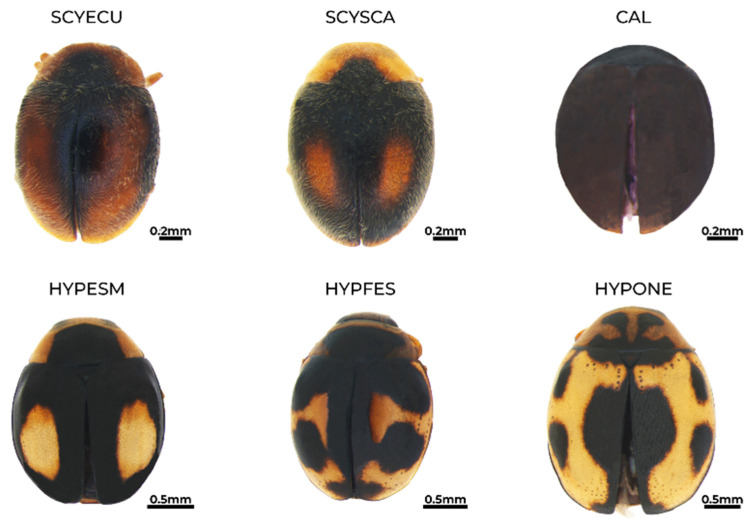
Habitus of *Scymnobius ecuadoricus* (SCYECU), *Scymnobius scalesius* (SCYSCA), *Calloeneis* sp. (CAL), *Hyperaspis esmeraldas* (HYPESM), *H. festiva* (HYPFES), and *H. onerata* (HYPONE).

*Scymnobius ecuadoricus* Gordon and González, 2002 [32] (Figure 7)
**Status in Galapagos:** Possibly native.
**Global distribution:** Ecuador and Peru [32,63].
**Distribution in the Galapagos islands:** San Cristóbal. This is the first report of the species on the archipelago.
**Ecosystems in San Cristóbal Island:** Urban green areas, permanent crops undergoing native forest regeneration, deciduous forests, and seasonal evergreen forests mixed with blackberry and supirosa.
**Diagnosis:** Size 1.5–2 mm. Oval-shaped body. Light brown head. The pronotum is light brown with a black semicircular basal spot that may vary in size or not be present. Light brown elytra with a black spot that covers half of the elytra. Light brown legs and antennae and abundant dorsal pubescens [32].
**Voucher specimens:** ECUADOR • ten specimens; Galapagos province, San Cristóbal Island, Baquerizo Beach trail; −0.888899, −79.607597; 40 m alt.; 18 and 19 July 2019; E. Peñaherrera-Romero and E. Cadena leg.; ZSFQ • eighteen specimens; Galapagos province, San Cristóbal Island, Baquerizo Beach trail; −0.888899, −79.607597; 40 m alt.; 23 and 25 July 2023; E. Peñaherrera-Romero, M.P. Rueda-Rodríguez, and E. Vélez-Darquea leg.; ZSFQ • one specimen; Galapagos province, San Cristóbal Island, Playa Mann and Environmental Interpretation Center; −0.894897, −89.60904; 9 m alt.; 15 August 2022; E. Peñaherrera-Romero, D. Die-Morejón, A. Guerrero-Campoverde and T. Guerrero-Molina leg.; ZSFQ • eleven specimens; Galapagos province, San Cristóbal Island, Playa Mann and Environmental Interpretation Center; −0.894897, −89.60904; 9 m alt.; 23 and 25 July 2023; E. Peñaherrera-Romero, M.P. Rueda-Rodríguez, and E. Vélez-Darquea leg.; ZSFQ • one specimen; Galapagos province, San Cristóbal Island, Puerto Baquerizo Moreno town; −0.904573, −89.611143; 13 m alt.; 21 July 2019; E. Peñaherrera-Romero and E. Cadena leg.; ZSFQ • one specimen; Galapagos province, San Cristóbal Island, Opuntias Beach trail; −0.932561, −89.584034; 40 m alt.; 23 July 2019; E. Peñaherrera-Romero and E. Cadena leg.; ZSFQ • one specimen; Galapagos province, San Cristóbal Island, Opuntias Beach trail; −0.932561, −89.584034; 40 m alt.; 26 July 2023; E. Peñaherrera-Romero, M.P. Rueda-Rodríguez, and E. Vélez-Darquea leg.; ZSFQ • two specimens; Galapagos province, San Cristóbal, Galapagos National Park patch 1; −0.883996, −89.539673; 365 m alt.; 29 July 2019; E. Peñaherrera-Romero and E. Cadena leg.; ZSFQ • one specimen; Galapagos province, San Cristóbal, Hacienda Tranquila, regeneration site; −0.886647, −89.539828; 392 m alt.; 17 February 2022; D.F. Cisneros-Heredia leg.; ZSFQ • one specimen; Galapagos province, San Cristóbal, Lobería Beach; −0.923923, −89.614669; 5 m alt.; 19 August 2022; E. Peñaherrera-Romero, D. Die-Morejón, A. Guerrero-Campoverde and T. Guerrero-Molina leg.; ZSFQ.

*Scymnobius scalesius* Gordon and González, 2002 [32] (Figure 7)
**Status in Galapagos:** Endemic.
**Global distribution:** This species can be found only in the Galapagos Archipelago [31,32].
**Distribution in the Galapagos islands:** Española, Fernandina, Floreana, Genovesa, Isabela, Marchena, Pinzón, Rábida, San Cristóbal, Santa Cruz, Santa Fé, and Santiago [31,32].
**Ecosystems in San Cristóbal Island:** Permanent crops (coffee and guava) undergoing native forest regeneration.
**Diagnosis:** Size 1.7 mm. Males with yellow heads with a dark brown fringe over their eyes and yellow a pronotum with a semicircular dark spot from its base to the middle of the pronotum without reaching the borders. Females with black heads and a black pronotum. Elytra is black with an elongated oval dark red spot and a yellow terminal margin. Dark yellow antennae, mouthparts, and legs. Pubescence of various sizes is distributed in the pronotum and elytra [32].
**Voucher specimens:** ECUADOR • two specimens; Galapagos province, San Cristóbal Island, Hacienda Tranquila, regeneration site; −0.886647, −89.539828; 392 m alt.; 14 and 15 July 2023; E. Peñaherrera-Romero, M.P. Rueda-Rodríguez, and E. Vélez-Darquea leg.; ZSFQ.

**Tribe Cryptognathini**

*Calloeneis* sp. (Figure 7)
**Status in Galapagos:** Undetermined.
**Global distribution:** Argentina, Bolivia, Brazil, Colombia, Guyana, Paraguay, Peru, Spain, Trinidad and Tobago, and Venezuela [53].
**Distribution in the Galapagos islands:** San Cristóbal. This is the first report of the genus on the archipelago and Ecuador.
**Ecosystems in San Cristóbal Island:** Permanent crops (coffee and guava) undergoing native forest regeneration and deciduous forests.
**Diagnosis:** Size <2 mm. Specimens with a mostly unmodified clypeus, nearly flat (or slightly descending) epipleuron, and prosternum less expanded than *Cryptognatha* [53]. Species-level identification requires examining male genitalia.
**Remarks:** To identify the species present in the Galapagos, further research, including genitalia morphology and colour variation, is required.
**Voucher specimens:** ECUADOR •one specimen; Galapagos province, San Cristóbal Island, Hacienda Tranquila, regeneration site; −0.886647, −89.539828; 392 m alt.; 30 July 2019; E. Peñaherrera-Romero and E. Cadena leg.; ZSFQ •one specimen; Galapagos province, San Cristóbal Island, Baquerizo Beach trail; −0.888899, −79.607597; 40 m alt.; 23 July 2023; E. Peñaherrerra-Romero, M.P. Rueda-Rodríguez, and E. Vélez-Darquea leg.; ZSFQ.

**Tribe: Hyperaspidini**

*Hyperaspis esmeraldas* Gordon and González, 2011 [50] (Figure 7)
**Status in Galapagos:** Possibly native.
**Global distribution:** Ecuador and Peru [50].
**Distribution in the Galapagos islands:** San Cristóbal and Santa Cruz. This is the first report of the species on the archipelago.
**Ecosystems in San Cristóbal Island:** Urban green areas, silvopasture, and deciduous forests.
**Diagnosis:** Size 2–3 mm. The body is slightly elongated at the head. Orange-yellow pronotum with a black semicircular spot in the centre. Elytra are black with two oval orange-yellow spots in the centre of each elytron. The legs, antennae, and mouthparts are yellow [50].
**Remarks:** We collected this species for the first time in 2019—the first observations in iNaturalist date back to 2008 in San Cristóbal [77].
**Voucher specimens:** ECUADOR • two specimens; Galapagos province, San Cristóbal Island, Baquerizo Beach trail; −0.888899,−79.607597; 40 m alt.; 18 July 2019; E. Peñaherrera-Romero and E. Cadena leg.; ZSFQ • three specimens; Galapagos province, San Cristóbal Island, Lobería Beach; −0.923923, −89.614669; 5 m alt.; 19 August 2022; E. Peñaherrera-Romero, D. Die-Morejón, A. Guerrero-Campoverde and T. Guerrero-Molina leg.; ZSFQ • one specimen; Galapagos province, San Cristóbal Island, Puerto Baquerizo Moreno town; −0.904573,−89.611143; 13 m alt.; 27 July 2019; E. Peñaherrera-Romero and E. Cadena leg.; ZSFQ •one specimen; Galapagos province, San Cristóbal Island, Hacienda Tranquila, agricultural lands; −0.887872, −89.539682; 384 m alt.; 4 February 2022; D.F. Cisneros-Heredia leg.; ZSFQ.

*Hyperaspis festiva* Mulsant, 1850 [73] (Figure 7)
**Status in Galapagos:** Possibly native.
**Global distribution:** This species is widely distributed across America, including the USA, Mexico, Honduras, Panama, Colombia, Brazil, Bolivia, Argentina, Antillas, and Granada [78]. The specimens reported herein are the first vouchered specimens of *H. festiva* from Ecuador.
**Distribution in the Galapagos islands:** San Cristóbal. This is the first report of the species on the archipelago.
**Ecosystems in San Cristóbal Island:** Urban green areas, seasonal evergreen forests mixed with blackberry and supirosa, and deciduous forests.
**Diagnosis:** Size 2–3 mm. Flattened and oval body shape; slightly more elongated on the posterior margin. The head is yellow in males and black in females. The pronotum is yellow with a black spot that covers almost the entire pronotum. Alytra are black with two irregular yellow spots that may be joined in some specimens. The border of elytra is yellow. Brown antennae, legs, and mouthparts [73].
**Voucher specimens:** ECUADOR • one specimen; Galapagos province, San Cristóbal Island, Galapagos National Park patch 1; −0.883996, −89.539673; 365 m alt.; 29 July 2019; E. Peñaherrera-Romero and E. Cadena leg.; ZSFQ • one specimen; Galapagos province, San Cristóbal Island, Playa Mann and Environmental Interpretation Center; −0.894897, −89.60904; 9 m alt.; 15 August 2022; E. Peñaherrera-Romero, D. Die-Morejón, A. Guerrero-Campoverde and T. Guerrero-Molina leg.; ZSFQ • one specimen; Galapagos province, San Cristóbal Island, Playa Mann and Environmental Interpretation Center; −0.894897, −89.60904; 9 m alt.; 25 July 2023; E. Peñaherrera-Romero, M.P. Rueda-Rodríguez, and E. Vélez-Darquea leg.; ZSFQ • one specimen; Galapagos province, San Cristóbal Island, Opuntias Beach trail; −0.932561, −89.584034; 40 m alt.; 4 August 2022; E. Peñaherrera-Romero, D. Die-Morejón, A. Guerrero-Campoverde and T. Guerrero-Molina leg.; ZSFQ.

*Hyperaspis onerata* (Mulsant, 1850) [73] (Figure 7)
**Status in Galapagos:** Possibly native.
**Global distribution:** Colombia and Ecuador [78,79]. Specimens reported herein are the first vouchered specimens of *H. onerata* from Ecuador.
**Distribution in the Galapagos islands:** Floreana, Isabela, San Cristóbal, and Santa Cruz. These are the first voucher specimens from the archipelago.
**Ecosystems in San Cristóbal Island:** Urban green areas and deciduous forests.
**Diagnosis:** Size 2–3 mm. Oblong body. Brownish yellow head. The pronotum has four fan-shaped black spots united at the base. The elytra are brownish yellow with a circular or oval spot extended at the suture towards the front and rear margins and there are two or one black spots on the sides. Legs, antennae, and mouthparts are reddish yellowish [73].
**Remarks:** We collected this species for the first time in 2019—the first observations in iNaturalist date back to 2008 in San Cristóbal [80].
**Voucher specimens:** ECUADOR • six specimens; Galapagos province, San Cristóbal Island, Playa Mann and Environmental Interpretation Center; −0.894897, −89.60904; 9 m alt.; 13 and 25 July 2023; E. Peñaherrera-Romero, M.P. Rueda-Rodríguez, and E. Vélez-Darquea leg.; ZSFQ • two specimens; Galapagos province, San Cristóbal Island, Lobería Beach; −0.923923, −89.614669; 5 m alt.; 15 August 2022; E. Peñaherrera-Romero, D. Die-Morejón, A. Guerrero-Campoverde and T. Guerrero-Molina leg.; ZSFQ • one specimen; Galapagos province, San Cristóbal Island, Puerto Baquerizo Moreno town; −0.904573, −89.611143; 13 m alt.; 21 July 2019; E. Peñaherrera-Romero and E. Cadena leg.; ZSFQ • one specimen; Galapagos province, San Cristóbal Island, Baquerizo Beach trail; −0.888899, −79.607597; 40 m alt.; 18 July 2019; E. Peñaherrera-Romero and E. Cadena leg.; ZSFQ • two specimens; Galapagos province, San Cristóbal Island, Baquerizo Beach trail; −0.888899, −79.607597; 40 m alt.; 23 July 2023; E. Peñaherrera-Romero, M.P. Rueda-Rodríguez, and E. Vélez-Darquea leg.; ZSFQ.

**Figure 8:**
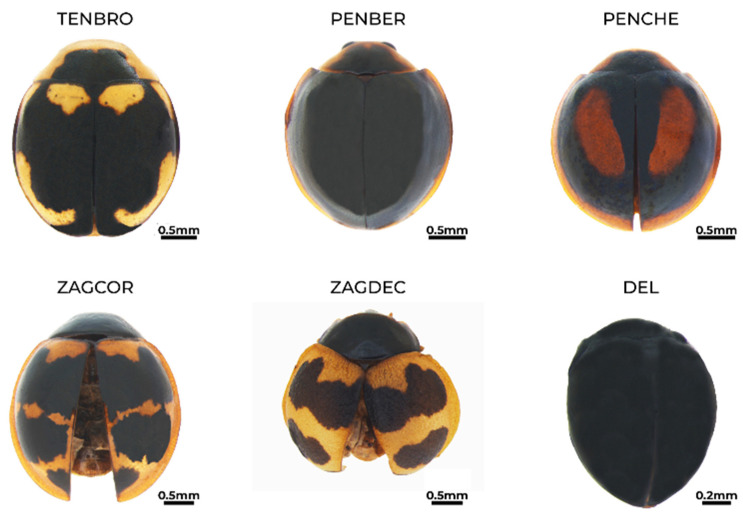
Habitus of *Tenuisvalvae bromelicola* (TENBRO), *Pentilia bernadette* (PENBER), *Pentilia chelsea* (PENCHE), *Zagreus cornejoi* (ZAGCOR), *Zagreus decempuctatus* (ZAGDEC), and *Delphastus* sp. (DEL).

*Tenuisvalvae bromelicola* (Sicard, 1925) [81] (Figure 8)
**Status in Galapagos:** Native.
**Global distribution:** Central America, Ecuador, and the Galapagos Archipelago [31].
**Distribution in the Galapagos islands:** Genovesa, Isabela, San Cristóbal, Santa Cruz, and Santiago [31].
**Ecosystems in San Cristóbal Island:** Urban green area and deciduous forests.
**Diagnosis:** Size 2–3 mm. Brown head. A yellowish pronotum with a trapezoid black spot. Elytra are black with four yellow spots; a black spot in the centre forms the shape of a butterfly or bat. Legs, antennae, and mouthparts are yellow. Without pubescence [81].
**Voucher specimens:** ECUADOR • one specimen; Galapagos province, San Cristóbal Island, Opuntias Beach trail; −0.932561, −89.584034; 40 m alt.; 23 of July 2023; E. Peñaherrera-Romero and E. Cadena leg.; ZSFQ • four specimens; Galapagos province, San Cristóbal Island, Playa Mann and Environmental Interpretation Center; −0.894897, −89.60904; 9 m alt.; 13 and 25 July 2023; E. Peñaherrera-Romero, M.P. Rueda-Rodríguez, and E. Vélez-Darquea leg.; ZSFQ • one specimen; Galapagos province, San Cristóbal Island, Baquerizo Beach trail; −0.888899, −79.607597; 40 m alt.; 18 July 2019; E. Peñaherrera-Romero and E. Cadena leg.; ZSFQ • seven specimens; Galapagos province, San Cristóbal Island, Baquerizo Beach trail; −0.888899, −79.607597; 40 m alt.; 23 and 25 July 2023; E. Peñaherrera-Romero, M.P. Rueda-Rodríguez, and E. Vélez-Darquea leg.; ZSFQ.

**Tribe: Pentiliini**

*Pentilia bernadette* Gordon, González, and Hanley, 2019 [54] (Figure 8)
**Status in Galapagos:** Possibly native.
**Global distribution:** Ecuador and Peru [54].
**Distribution in the Galapagos islands:** San Cristóbal. This is the first report of the species on the archipelago.
**Ecosystems in San Cristóbal Island:** Permanent crops (coffee and guava) undergoing native forest regeneration.
**Diagnosis:** Size 2–3 mm. Head yellow. The pronotum is blackish brown with a yellow edge. Elytra are blackish brown with a narrow yellow lateral edge. Dark orange antennae, legs, and mouthparts. Coarse punctures in all its body, head, and pronotal punctures are separated by the same measurement of the puncture or less and elytral punctures larger than on pronotum and separated by more than the measurement of the punctures [54].
**Remarks:** The description of *P. bernadette* states that “The black elytra with a narrow, lateral, yellow border distinguish this species from all other species of *Pentilia* except *P. traci*. The latter species is Brazilian with a wider lateral border on each elytron and has different male genitalia”. Although the genitalia of our specimens were not examined; their colouration matches that of *P. bernadette*. We excluded *P. traci* because it is improbable that a species known only from a locality in the interior of the state of Santa Catarina, southern Brazil, could have reached the Galapagos, even as a non-native species. In contrast, *P. bernadette* is known from the Pacific coast of northern Peru and Ecuador.
Peck [31] reported an undescribed species of *Pentilia* on four islands in the Galapagos, including San Cristóbal, but there was no description or diagnostic details. It is possible that it could correspond to this species.
**Voucher specimens:** ECUADOR • one specimen; Galapagos province, San Cristóbal Island, Hacienda Tranquila, regeneration site; −0.886647, −89.539828; 392 m alt.; 17 February 2022; D.F. Cisneros-Heredia leg.; ZSFQ.

*Pentilia chelsea* Gordon, González, and Hanley, 2019 [54] (Figure 8)
**Status in Galapagos:** Possibly native.
**Global distribution:** British Guyana, Colombia, and Ecuador [54].
**Distribution in the Galapagos islands:** San Cristóbal and Santa Cruz. This is the first report of the species on the archipelago.
**Ecosystems in San Cristóbal Island:** Permanent crops (coffee and guava) undergoing native forest regeneration, silvopasture, and seasonal evergreen forests mixed with blackberry and supirosa.
**Diagnosis:** Size 2–3 mm. Yellow head. The pronotum is yellow with a black semicircular spot. Elytra are black with two elongated oval yellow spots and yellow lateral edges. Legs, antennae, and mouthparts are yellow. No pubescence [54].
**Remarks:** The description of *P. chelsea* states that “In spite of the color variation in the elytral maculae, this species may be identified by dorsal color pattern”. Although genitalia were not examined in our specimens, the colouration matches that of *P. chelsea*. Species that are similar to *P. chelsea* include *P. mable* and *P. ernestine*. According to Gordon et al. [54], “*P. mable* somewhat resembles *P. chelsea* but is smaller and has a mostly pale elytron bordered with dark brown”; furthermore, it is known from the Eastern Cordillera of the Andes of northern Colombia. Interestingly, genitalia do not help differentiate *P. chelsea* and *P. ernestine*, and Gordon et al. [54] stated that “*Pentilia ernestine* has male genitalia very similar to those of *P. chelsea*, but both are maintained as valid based on differences in the dorsal color pattern”. Furthermore, *P. ernestine* is known from the Central Cordillera of the Andes of northern Colombia, while *P. chelsea* is known from the Pacific coasts of Ecuador and northern Peru.
Peck [31] reported an undescribed species of *Pentilia* on four islands in the Galapagos, including San Cristóbal, but there was no description or diagnostic details. It is possible that it could correspond to this species.
**Voucher specimens:** ECUADOR • two specimens; Galapagos province, San Cristóbal Island, Galapagos National Park patch 2; −0.88374, −89.539804; 365 m alt.; 1 August 2019; E. Peñaherrera-Romero and E. Cadena leg.; ZSFQ • one specimen; Galapagos province, San Cristóbal Island, Hacienda Tranquila, regeneration site; −0.886647, −89.53982; 392 m alt.; 30 July 2019; E. Peñaherrera-Romero and E. Cadena leg.; ZSFQ • one specimen; Galapagos province, San Cristóbal Island, Hacienda Tranquila, regeneration site; −0.886647, −89.53982; 392 m alt.; 17 August 2022; E. Peñaherrera-Romero, D. Die-Morejón, A. Guerrero-Campoverde and T. Guerrero-Molina leg.; ZSFQ • one specimen; Galapagos province, San Cristóbal Island, Hacienda Tranquila, agricultural lands; −0.887872, −89.539682; 384 m alt.; 2 August 2022; D.F. Cisneros-Heredia leg.; ZSFQ.

**Tribe Chilocorini**

*Zagreus constantini* (González, 2015) [82]
**Status in Galapagos:** Possibly native.
**Global distribution:** Ecuador [82].
**Distribution in the Galapagos islands:** San Cristóbal. This is the first report of the species on the archipelago.
**Ecosystems in San Cristóbal Island:** Seasonal evergreen forests mixed with blackberry and supirosa.
**Diagnosis:** Size 3–4 mm. Elongated oval shape. The head and pronotum are yellowish brown. Yellow elytra with five rounded black spots of different sizes. Legs, antennae, abdomen, and mouthparts are dark brown [82].
**Remarks:** Many observed specimens were not collected during our surveys.
**Voucher specimens:** ECUADOR • one specimen; Galapagos province, San Cristóbal Island; Galapagos National Park patch 1; −0.883996, −89.539673; 365 m alt.; 29 July 2019; E. Peñaherrera-Romero and E. Cadena leg.; ZSFQ.

*Zagreus cornejoi* González and Almeida, 2017 [51] (Figure 8)
**Status in Galapagos:** Possibly native
**Global distribution:** Ecuador [51].
**Distribution in the Galapagos islands:** San Cristóbal. This is the first report of the species on the archipelago.
**Ecosystems in San Cristóbal Island:** Urban green area and deciduous forests.
**Diagnosis:** Size 3–4 mm. Elongated oval shape. The body and part of the pronotum are yellowish, the head is black, and each elytron is orange brown with three irregular transverse black stripes with size variations between individuals. Legs and antennae are yellow with black endings [51].
**Voucher specimens:** ECUADOR • three specimens; Galapagos province, San Cristóbal Island, Playa Mann and Environmental Interpretation Center; −0.894897, −89.60904; 9 m alt.; 16 July 2019; E. Peñaherrera-Romero and E. Cadena leg.; ZSFQ • two specimens; Galapagos province, San Cristóbal Island, Playa Mann and Environmental Interpretation Center; −0.894897, −89.60904; 9 m alt.; 13 July 2023; E. Peñaherrera-Romero, M.P. Rueda-Rodríguez, and E. Vélez-Darquea leg.; ZSFQ • two specimens; Galapagos province, San Cristóbal Island; Opuntias Beach trail; −0.932561, −89.584034; 40 m alt.; 24 July 2019; E. Peñaherrera-Romero and E. Cadena leg.; ZSFQ • five specimens; Galapagos province, San Cristóbal Island, Opuntias Beach trail; −0.932561, −89.584034; 40 m alt.; 16 August 2022; E. Peñaherrera-Romero, D. Die-Morejón, A. Guerrero-Campoverde and T. Guerrero-Molina leg.; ZSFQ • one specimen; Galapagos province, San Cristóbal Island, Baquerizo Beach trail; −0.888899, −79.607597; 40 m alt.; 18 July 2019; E. Peñaherrera-Romero and E. Cadena leg.; ZSFQ • five specimens; Galapagos province, San Cristóbal Island, Baquerizo Beach trail; −0.888899, −79.607597; 40 m alt.; 23 July 2023; E. Peñaherrera-Romero, M.P. Rueda-Rodríguez, and E. Vélez-Darquea leg.; ZSFQ • one specimen; Galapagos province, San Cristóbal Island, Lobería Beach; −0.923923, −89.614669; 5 m alt.; 19 August 2022; E. Peñaherrera-Romero, D. Die-Morejón, A. Guerrero-Campoverde and T. Guerrero-Molina leg.; ZSFQ.

*Zagreus decempunctatus* (Weise, 1893) [83] (Figure 8)
**Status in Galapagos:** Possibly native.
**Global distribution:** Ecuador and Peru [83,84].
**Distribution in the Galapagos islands:** San Cristóbal. This is the first report of the species on the archipelago.
**Ecosystems in San Cristóbal Island:** Seasonal evergreen forests mixed with blackberry and supirosa and deciduous forests.
**Diagnosis:** Size 3–4 mm. Semicircular. Light to dark brown head. The pronotum is black with yellowish margins. Elytra have five circular or oval black spots that do not join or touch the margin. The rest of the elytra are dark yellow and atypically red. Legs, antennae, and mouthparts are yellowish brown, without pubescence [83].
**Voucher specimens:** ECUADOR • two specimens; Galapagos province, San Cristóbal Island, Baquerizo Beach trail; −0.888899, −79.607597; 40 m alt.; 18 July 2019; E. Peñaherrera-Romero and E. Cadena leg.; ZSFQ • one specimen; Galapagos province, San Cristóbal Island, Opuntias Beach trail; −0.932561, −89.584034; 40 m alt.; 23 July 2019; E. Peñaherrera-Romero and E. Cadena leg.; ZSFQ • one specimen; Galapagos province, San Cristóbal Island, Galapagos National Park patch 1; −0.883996, −89.539673; 365 m alt.; 29 July 2019; E. Peñaherrera-Romero and E. Cadena leg.; ZSFQ.

**Tribe: Sticholotidini**

*Delphastus* sp. (Figure 8)
**Status in Galapagos:** Undetermined.
**Global distribution:** The genus occurs in the Western Hemisphere [52].
**Distribution in the Galapagos islands:** This is the first report of the genus on the archipelago.
**Ecosystems in San Cristóbal Island:** Permanent crops (coffee and guava) undergoing native forest regeneration and deciduous forests.
**Diagnosis:** Size <2 mm. Species of this genus show oblong and slightly convex black or very dark brown bodies, well-developed antennae, narrow epipleurae, and anterior tibia and tarsi completely concealed by prosternal depression. The elytron is non-maculate and appears impunctate, and the prosternum has scattered, setose punctures [85]. Species-level identification requires a better understanding of colour variation and examination of genitalia.
**Remarks:** Few specimens are available, and further research, including genitalia examination, is required to identify the species present in the Galapagos.
**Voucher specimens:** ECUADOR • five specimens; Galapagos province, San Cristóbal Island, Hacienda Tranquila, regeneration site; −0.886647, −89.539828; 392 m alt.; 17 August 2022; E. Peñaherrera-Romero, D. Die-Morejón, A. Guerrero-Campoverde and T. Guerrero-Molina leg.; ZSFQ • one specimen; Galapagos province, San Cristóbal Island, Baquerizo Beach trail; −0.888899, −79.607597; 40 m alt.; 23 July 2023; E. Peñaherrerra-Romero, M.P. Rueda-Rodríguez, and E. Vélez-Darquea leg.; ZSFQ.

## 4. Discussion

Our study reveals that the ladybird beetle community on San Cristóbal Island is much more complex than has been reported in previous studies [27,28,29,30,31,32,33,34,35]. We recorded all endemic and native species on the island previously reported by Peck [31]. Most of these species, except for *Cycloneda sanguinea*, are rare to uncommon. In addition, we reported a large number of species previously unreported for the island, almost doubling the number of known species—many of these species were also recorded for the first time in the archipelago or even the country. These results suggest that the ladybird beetle community in the Galapagos is underestimated and underscore the importance of continued taxonomic and ecological research on the islands. Further studies across other islands of the Galapagos Archipelago are likely to reveal greater species richness.

The increase in the species richness of ladybird species on San Cristóbal Island may be attributed to the reduced previous sampling effort allocated to this island and the fact that most entomological surveys were concentrated in the lowland dry ecosystems of San Cristóbal. For example, Peck’s team [31] invested 465 person days surveying San Cristóbal Island, with 17.6% of that effort in the forested highlands. In contrast, they invested 5045 person days in Santa Cruz Island, including 41% in the forested highlands. Also, all their efforts were primarily focused on natural ecosystems, with just 6.3–12.9% invested in urban and agricultural areas. In comparison, our survey efforts were equally distributed across all four studied habitats, combining human-made and natural ecosystems. The use of information obtained through participatory science programmes, such as iNaturalist, also allowed us to identify records from time periods outside our study. Participatory science was also crucial to engaging island society, especially young students, in generating data within their communities [86]. However, there is a clear bias in this type of recording towards larger, more conspicuously coloured species. Also, our focused efforts on a single island and a specific clade of Coleoptera likely enabled us to uncover a greater diversity [87]. 

It is possible that some of the species reported for the first time in this study, and identified as possibly native, are non-native species that have recently arrived through human-mediated processes. Ladybird beetles can easily arrive on the islands inadvertently carried by air or sea in industrial, agricultural, or food cargo or visitors’ luggage. Several non-native species that have arrived in the Galapagos in recent years have done so by this means of arrival, which has become increasingly common as travel to the islands increases [56]. Further studies are required to confirm the zoogeographic status of the species herein reported as possible native [15,31,88]. This challenge is compounded by the absence of baseline information about native invertebrates in the Galapagos, which complicates the identification of introduced species [57,88]. Historically, much attention has been directed towards vertebrate species, making it challenging to develop effective management responses for non-native invertebrates due to limited information [15,56,57,88,89].

The presence of two expanding non-native species, *Cheilomenes sexmaculata* and *Paraneda guticollis*, raises concerns about the impact of non-native species on the native and endemic fauna of the Galapagos. The widespread distribution of *C. sexmaculata* across various ecosystems, including agricultural and urban areas, suggests that this species is well adapted to the diverse habitats of San Cristóbal Island and could potentially disrupt local ecological balances. This species has recently spread across America and can be considered a potentially dangerous invasive species. The possible expansion of *P. guticollis* into natural habitats, as indicated by its abundance in urban areas and presence in deciduous forests and agricultural lands, suggests that it may also pose a growing threat to native species and local ecological processes. Invasive ladybird beetles can have substantial negative impacts on native biodiversity and agriculture. They may prey on non-target arthropods, alter invertebrate assemblages, outcompete or displace native coccinellids through competition and predation, and feed on or damage agricultural products [47,90,91,92,93,94]. The impacts of non-native invertebrates are often overlooked until they cause significant issues [95]. The presence of non-native Coccinellidae could have substantial socioeconomic effects on the islands, particularly in agriculture and native ecosystems [23,96,97]. Therefore, ongoing research is crucial due to the complex nature of these ecosystems and the many factors influencing ladybird diversity and abundance [94,98,99].

Ladybirds typically exhibit a functional response as predators, characterised by their ability to adapt their predation to their prey availability over time, which makes them successful generalists [100]. *Novius cardinalis* shows a modified response, making it an effective pest controller due to its less aggressive behaviour toward other prey than other ladybird species, which can be considered a specialist species. This behaviour likely contributes to its non-hyperabundance and minimises competition with native ladybirds [101,102]. However, the overabundance of non-native ladybird species could be already problematic [94]. The abundant *Cheilomenes sexmaculata* and *Paraneda guticollis* could already produce habitat compression, isolating native species to reduced spaces [23,62,103].

The rarity and restricted distribution of endemic species, like *Psyllobora bisigma* and *Scymnobius scalesius*, suggest that these species may be particularly vulnerable to habitat changes and require targeted conservation efforts. However, the absence of certain species previously recorded on the islands may be due to methodological biases or natural rarity rather than population declines or changes in distribution. Three of the four species we did not record are small and may be less recorded during visual surveys. Given this, further research will require the use of a greater diversity of sampling methodologies, such as beating trays and sweep nets. In addition, as several non-native species have conspicuous colouration, the combined use of field surveys with participatory science and artificial intelligence programmes could help maximise their detectability in the early stages of their introduction [104,105]. Interestingly, at least two species unrecorded during our study are non-native (*Coccidophilus* sp. and *Hippodamia convergens*) and may have populations reduced in size or restricted to specific agricultural areas. Despite being a medium-sized and colourful species, the absence of records of *Hippodamia convergens* in iNaturalist suggests that the species has not expanded on the island.

Island ecosystems, with their unique biodiversity and physical environments, influence the specialisation of coccinellids [23,106,107]. Tropical oceanic islands are particularly vulnerable to non-native insect invasions due to their isolation from continental landmasses. This vulnerability can lead to reduced competitiveness, disharmony between functional groups, reduced diversity, and loss of adaptability and resistance to predators and disease [2,108,109,110]. Non-native species often arrive through human-mediated means, sometimes deliberately and sometimes accidentally [87,111,112]. Globalisation, driven by trade and tourism, represents the primary pathway for these invasions [113]. However, the lack of detailed information on the arrival dynamics of these species hinders our understanding of their frequency and pathways [114,115]. If some of the species reported for the first time in the Galapagos in this study are non-native, they may already play significant roles in the ecological interactions of the island. In other locations, studies have documented biodiversity loss and agricultural impacts due to non-native species at both ecological and economic levels [23,47,90,92,93,116,117]. Although the interspecific interactions among ladybird beetles on San Cristóbal Island remain unknown, the impacts of non-native ladybird beetle species on native populations have been well documented elsewhere. Aggressive colonising species of Coccinellidae have been shown to displace native species in continental ecosystems [23,105,106,118]. In addition, the insular syndrome, where species that have evolved in isolation are disadvantaged, may further affect native ladybirds [96].

Our study shows that agroecological systems undergoing habitat regeneration in San Cristóbal are crucial for ladybird beetle populations, especially endemic species [119]. Continued research in Galapagos agroecosystems is essential, as our understanding of these systems is limited. Variables such as natural areas surrounding farms, monocultures, non-native species, and urbanisation expansion can affect insect richness and abundance within agroecosystems [94,98,99].

Our study provides critical insights into the composition, distribution, and potential threats to the ladybird beetle community on San Cristóbal Island. These findings have important implications for the conservation and management of biodiversity in the Galapagos islands and emphasise the need for continued research and monitoring to ensure the protection of these unique ecosystems.

## Figures and Tables

**Table 1:** Information for the localities explored during our surveys for ladybird beetles in the San Cristóbal Island, Galapagos Archipelago, Ecuador.

Locality	Coordinates	Elevation	Habitat	Ecosystem
Encañada ravine	−0.906, −89.611	16 m	Urban green area	Urban
Playa Mann and Environmental Interpretation Center	−0.895, −89.609	12 m		
Puerto Baquerizo Moreno town	−0.904, −89.609	20 m		
Baquerizo Beach trail	−0.888, −89.605	31 m	Deciduous forest	Deciduous forest
Cerro Tijeretas trail	−0.891, −89.609	30 m		
Lobería Beach	−0.924, −89.612	9 m		
Opuntias Beach trail	−0.938, −89.549	90 m		
Hacienda Tranquila, agricultural lands	−0.890, −89.538	401 m	Silvopasture	Agricultural
Hacienda Tranquila, regeneration site	−0.886, −89.539	388 m	Permanent crops (coffee and guava) undergoing native forest regeneration	
Hacienda Tranquila, Risco de los Petreles	−0.887, −89.531	502 m	Silvopasture	
Galapagos National Park patch 1	−0.882, −89.536	395 m	Seasonal evergreen forest mixed with blackberry (*Rubus niveus*) and supirosa (*Lantana camara*)	Seasonal evergreen forest
Galapagos National Park patch 2	−0.883, −89.543	339 m		

**Table 2:** Relative abundance of the 19 documented species with their zoogeographic status (E = endemic, N = native, pN = possibly native, pNN = possibly non-native, NN = non-native, and U = undetermined) and their relative abundance and number of individuals (between parentheses) by ecosystem (U = urban, A = agricultural, D = deciduous forest, and S = seasonal evergreen forest). Species reported for the first time on the Galapagos islands in this study are marked with an asterisk.

Tribe	Species	Status	Ecosystem			
			U	A	D	S
Stethorini	*Stethorus* sp. *	U	0	0.002 (1)	0	0.008 (1)
Coccinellini	*Cheilomenes sexmaculata*	NN	0.097 (67)	0.136 (56)	0.015 (2)	0.042 (5)
	*Cycloneda sanguinea*	N	0.288 (198)	0.779 (321)	0.408 (53)	0.975 (116)
	*Paraneda guticollis*	pNN	0.570 (392)	0.010 (4)	0.015 (2)	0
	*Psyllobora bisigma*	E	0	0.029 (12)	0.008 (1)	0
Noviini	*Novius cardinalis*	NN	0.001 (1)	0.002 (1)	0	0
Scymnini	*Scymnobius scalesius*	E	0	0.005 (2)	0	0
	*Scymnobius ecuadoricus* *	pN	0.017 (12)	0.002 (1)	0.246 (32)	0.017 (2)
Cryptognathini	*Calloeneis* sp. *	U	0	0.002 (1)	0.008 (1)	0
Hyperaspidini	*Hyperaspis esmeraldas* *	pN	0.001 (1)	0.012 (5)	0.038 (5)	0
	*Hyperaspis festiva* *	pN	0.001 (1)	0	0.015 (2)	0.008 (1)
	*Hyperaspis onerata*	pN	0.01 (7)	0	0.038 (5)	0
	*Tenuisvalvae bromelicola*	N	0.006 (4)	0	0.069 (9)	0
Pentiliini	*Pentilia bernadette* *	pN	0	0.002 (1)	0	0
	*Pentilia chelsea* *	pN	0	0.007 (3)	0	0.017 (2)
Chilocorini	*Zagreus constantini* *	pN	0	0	0	0.008 (1)
	*Zagreus cornejoi* *	pN	0.007 (5)	0	0.108 (14)	0
	*Zagreus decempunctatus* *	pN	0	0	0.023 (3)	0.008 (1)
Sticholotidini	*Delphastus* sp. *	U	0	0.01 (4)	0.008 (1)	0

## Data Availability

All data supporting this study’s findings are available in the main text and the following depositories: Zenodo.
